# Identification of QTLs and new candidate genes affecting ear shank length via BSA-seq and transcriptomic analysis in maize

**DOI:** 10.3389/fpls.2026.1768852

**Published:** 2026-02-09

**Authors:** Hongzhou An, Kuiying Li, Xiaolan Liu, Yuhua Wu, Jianhan He, Yanbing Wang, Zengyu Gao

**Affiliations:** 1 Hebei Key Laboratory of Crop Genetics and Breeding, Institute of Cereal and Oil Crops, Hebei Academy of Agriculture and Forestry Sciences, Shijiazhuang, China; 2 Biotechnology and Nuclear Technology Research Institute, Sichuan Academy of Agricultural Sciences, Chengdu, China

**Keywords:** BSA-seq, candidate genes, ear shank length, maize, transcriptomic analysis

## Abstract

**Introduction:**

The ear shank, a short branch connecting the stalk and the ear, represented a key agronomic trait influenced both yield and plant architecture in maize, yet the molecular mechanism remained not fully understood.

**Methods:**

In this study, BSA-seq was performed using an F_2_ population for two extreme bulks derived from the cross between WL134 and L135. Additionally, transcriptomic analysis and gene annotation were carried out to refine the association interval of ear shank length and identify crucial genes.

**Results and Discussion:**

A total of 14 QTLs for ear shank length were detected, which included 334 non-synonymous mutants, synonymous mutants and frameshift mutant genes. Among these loci, five were known to be associated with ear shank length, while nine were newly identified. 3,460 differentially expressed genes (DEGs) were screened through RNA-seq analysis of the ear shank at the silking stage in both parents. Thirteen new candidate genes were identified through the combined analysis of BSA-seq and RNA-seq, as well as gene function annotation and gene expression analysis. Based on functional predictions, the candidate genes *Zm00001eb023400*, *Zm00001eb023420* and *Zm00001eb050490*, which encoded lytic transglycosylases, significantly associated with cell wall remodeling and degradation. The candidate genes *Zm00001eb282410* and *Zm00001eb282430* enriched the phenylpropanoid biosynthesis pathway and played important roles in the formation of the maize ear shank. These findings provided a foundation for understanding the molecular mechanisms regulating ear shank length in maize.

## 1 Introduction

As one of the most important grain crops worldwide, maize yield potential is closely associated with its plant architecture (Wen et al., 2025). The ear shank, a key structure connecting the stalk and the ear, is responsible for water and nutrient transport, as well as temporary storage and remobilization of carbohydrates during the grain-filling stage. Its length directly influences ear position, pollination efficiency and harvest index (He et al., 2005; Sun et al., 2022). Among maize varieties, average ear shank length typically ranges from 8 to 15 cm. A shorter ear shank often results in tight husk coverage, which affects ear dehydration. Conversely, an excessively long ear shank may increase kernel damage during mechanical harvesting (Sun et al., 2014; Wang et al., 2021). Therefore, elucidating the genetic mechanisms underlying ear shank length is of great significance for optimizing maize plant architecture and enhancing yield potential.

Ear shank length was a quantitative trait controlled by multiple genes, with a narrow-sense heritability of 72.2% (Hansen et al., 2001). Six stable major effect quantitative trait loci (QTLs) for ear shank length were identified in three genotyped recombinant inbred line populations (Liu M et al., 2021). Among these, QTLs on chromosomes 1 and 2 overlapped genetically with regions previously associated with ear and husk related traits reported by Xiao and Cui (Xiao et al., 2016; Cui et al., 2018). Sun et al. (2022) employed genome-wide association studies (GWAS) to analyze the genetic basis of ear shank length, identifying five significant QTLs. The gene *ZmELF3.1* regulated plant height, leaf number, internode number and ear shank length. Loss function of *ZmELF3.1* increased both plant height and ear shank length (Zhao et al., 2023). Knockout of the jasmonic acid related genes *OPR7* and *OPR8* resulted in elongation of the ear shank and stimulated the development of female reproductive buds at each node (Yan et al., 2012). *Cyclin11*, which showed peak expression in ear shanks 15 days after silking, played a crucial role in regulating ear shank length (Liang et al., 2022). Traditional QTL mapping methods have identified several QTLs associated with ear shank traits. However, precise gene cloning remains challenging due to low resolution and large confidence intervals.

In recent years, bulk segregation analysis sequencing (BSA-seq) had facilitated rapid and efficient gene mapping through the construction of segregating populations, selection of extreme phenotypic bulks and integration of high-throughput sequencing (Yang et al., 2025). Concurrently, RNA-seq technology revealed gene expression patterns at the transcriptome level, thereby providing expression evidence that aided in prioritizing candidate genes (Li et al., 2025). The combined analysis of BSA-seq and RNA-seq had become an effective strategy for gene mapping, achieving significant advancements in areas such as salt tolerance (Zhu et al., 2023), plant height (Gao et al., 2022), *Meloidogyne graminicola* resistance (Yang et al., 2025) and seed storability (Zhou et al., 2025). Nevertheless, this strategy has yet to be applied to explore the genetic mechanisms that regulating ear shank length.

The identification of genes that regulate ear shank length remains a critical area of focus in maize research. Consequently, it is essential to utilize new germplasm resources and populations to discover additional genes/QTLs associated with ear shank length, thereby establishing a foundation for a deeper understanding of the genetic mechanisms regulating maize plant architecture and yield. In this study, we constructed a segregating population by crossing the long ear shank inbred line L135 with the relatively short ear shank inbred line WL134. Through integrated BSA-seq and RNA-seq analysis, we performed genetic dissection of ear shank length to map major effect QTLs. Our objective was to identify novel candidate genes that regulate ear shank length, thus providing a basis for enhancing our comprehension of the regulatory mechanisms associated with this trait.

## 2 Materials and methods

### 2.1 Plant materials

Two corn varieties were used for hybridization in this study. The relatively short ear shank inbred line WL134 developed by our research team, featuring high comprehensive combining ability as the female parent. The long ear shank line L135 derived from a double haploid (DH) population constructed through the cross between WL134 and D7 inbred line with excellent resistance developed by our research team as the male parent. F_2_ generations were harvested through self-pollination after F_1_ planting. The F_2_ mapping population and parents were planted during the summer of 2024 at Dishang Experimental Station (114°43′7.928″ E, 37°56′25.800″ N) of the Institute of Cereal and Oil Crops, Hebei Academy of Agriculture and Forestry Sciences. Parents were planted 2 rows and F_2_ population planted 25 rows. The plants were grown with a row length of 6 m, row spacing of 0.5 m, under standardized production conditions for planting and management. At maturity, the ear shank length (actual length from the bottom of the ear to the stem attachment point) was measured with band tapes repeating three times. Due to the curved structure of the ear shank, the tape was kept closely adhered to the ear shank during measurement to minimize errors.

### 2.2 The construction of the extreme population

Through field cultivation and measurement of ear shank length, 30 plants with extremely short ear shanks were selected from the F_2_ population to form the short ear shank bulk (S-pool), and 30 plants with extremely long ear shanks were selected to form the long ear shank bulk (L-pool). The corresponding individual plants were numbered, and leaf samples were collected from each for preliminary mapping of the maize ear shank length genes.

### 2.3 BSA-seq analysis

BSA-seq was used to identify the genes regulating ear shank length in the F_2_ population. We selected 30 plants that were extremely long and short ear shank length to create an extreme population. Leaf samples were collected from three individuals of each parental line, as well as from the 30 plants in each of the extreme ear shank bulks (S-pool and L-pool). These samples were submitted to SMART GENOMICC (Qingdao, China) for BSA-seq analysis. The sequencing depth for the parental lines was set at 20×, while that for the offspring pools was set at 30×.DNAsecure Plant Kit (TIANGEN) was used for DNA extraction from plant tissues. The DNA quality was checked using NanoDrop 2000 spectrophotometer (Thermo Fischer Scientific), Agarose gel electrophoresis and Qubit fluorometer (Invitrogen). Qualified DNA samples were randomly broken into 350bp fragments by a Covaris crusher. Sequencing libraries were constructed following the segmentation of DNA through end repair, addition of adenine to the 3’ ends, adapter ligation and PCR amplification. Once the library passed quality checks, sequencing was performed using the DNBSEQ-T7 platform. The default parameters of fastp (Chen et al., 2018) were used for quality control, unqualified reads were filtered, and the clean reads obtained were used for subsequent analysis. Clean reads were aligned to the reference genome sequences of the B73 genome (https://download.maizegdb.org/Zm-B73-REFERENCE-NAM-5.0/) using Sentieon software (Pei et al., 2021). The mapping results were sorted and deduplicated using Samtools (Li et al., 2009). SNPs and InDels were detected and annotated using Sentieon (Pei et al., 2021) and ANNOVAR software (Wang et al., 2010). The SNP/InDels were selected with read depth ≥ 4, RMS Mapping Quality ≥ 40 and Genotype Qualit ≥ 5. The association analysis was conducted with ΔSNP/InDel index (Fekih et al., 2013) and G′-value (Magwene et al., 2011). The overlapped regions based on the above two methods were considered candidate regions for ear shank length.

### 2.4 Transcriptome sequencing

The collected samples from three replicated plants were stored in refrigerator at -80°C. The ear shank at silking stage of WL134 and L135 were used for transcriptome sequencing. DNA library generation and RNA-seq high throughput sequencing were performed by SMART GENOMICC (Qingdao, China). The total RNA was extracted from the samples using standard extraction methods. RNA quality control was assessed with the Agilent 2100 bioanalyzer. The mRNA with a polyadenylic acid tail was enriched by connecting oligothymidine magnetic beads, and then the obtained mRNA was randomly interrupted with divalent cations in NEB fragmentation buffer (Parkhomchuk et al., 2009). mRNA quality, including the mRNA concentration and fragment size, was tested by using Qubit2.0 and Agilent 2100. A total of 6 qualified libraries were sequenced on the Illumina Novaseq platform HiSeqTM 2500 (Illumina, San Diego, CA, USA), and 150 bp paired-end reads were generated.

Raw sequence reads were processed using fastp (Chen et al., 2018) for quality control to filter the unqualified reads with default parameters. The clean reads were mapped to a B73 reference of maize genome (https://download.maizegdb.org/Zm-B73-REFERENCE-NAM-5.0/) using hisat2 (Mortazavi et al., 2008). The number of fragments per kilobase of transcript per million mapped reads (FPKM) value for each gene was calculated using Featurecounts (Liao et al., 2014). Differential expression analysis between the two comparative combinations was performed using DESeq2 software (Love et al., 2014). The method of Benjamini and Hochberg was used to adjust the resulting P-values to control for false discovery rates. Genes with adjusted P-values <0.05 were classified by DESeq2 as differentially expressed. Gene Ontology (GO) divides the functions of genes into three parts: cellular component (CC), molecular function (MF) and biological process (BP). The ClusterProfiler software was used to perform GO functional enrichment analysis and Kyoto Encyclopedia of Genes and Geno (KEGG) pathway enrichment analysis on the differential gene sets (Wu et al., 2021). Padj less than 0.05 was used as the threshold of significant enrichment.

### 2.5 Real-time PCR

Total RNA was extracted from the ear shanks at silking stage using an RNA pure plant kit (TIANGEN, Beijing, China). The first strand of cDNA was synthesized according to the instructions for the HiScript^®^III RT SuperMix for qPCR (Vazyme, Nanjing, China). Subsequently, qRT‐PCR was performed using the ChamQ SYBR Color qPCR Master Mix (Vazyme, Nanjing, China). qRT-PCR was performed on CFX384 Real-Time System (BIO-RAD, United States). The maize Tubulin gene was used as the internal control. The 2^–ΔΔCT^ (Livak and Schmittgen, 2001) quantitative analysis method was used to calculate the relative expression level. The primers used in this study are listed in **Supplementary Table S1**.

## 3 Result

### 3.1 Phenotypic assessment of ear shank length in parental lines and construction of BSA-seq extreme mapping populations

**Figure 1:**
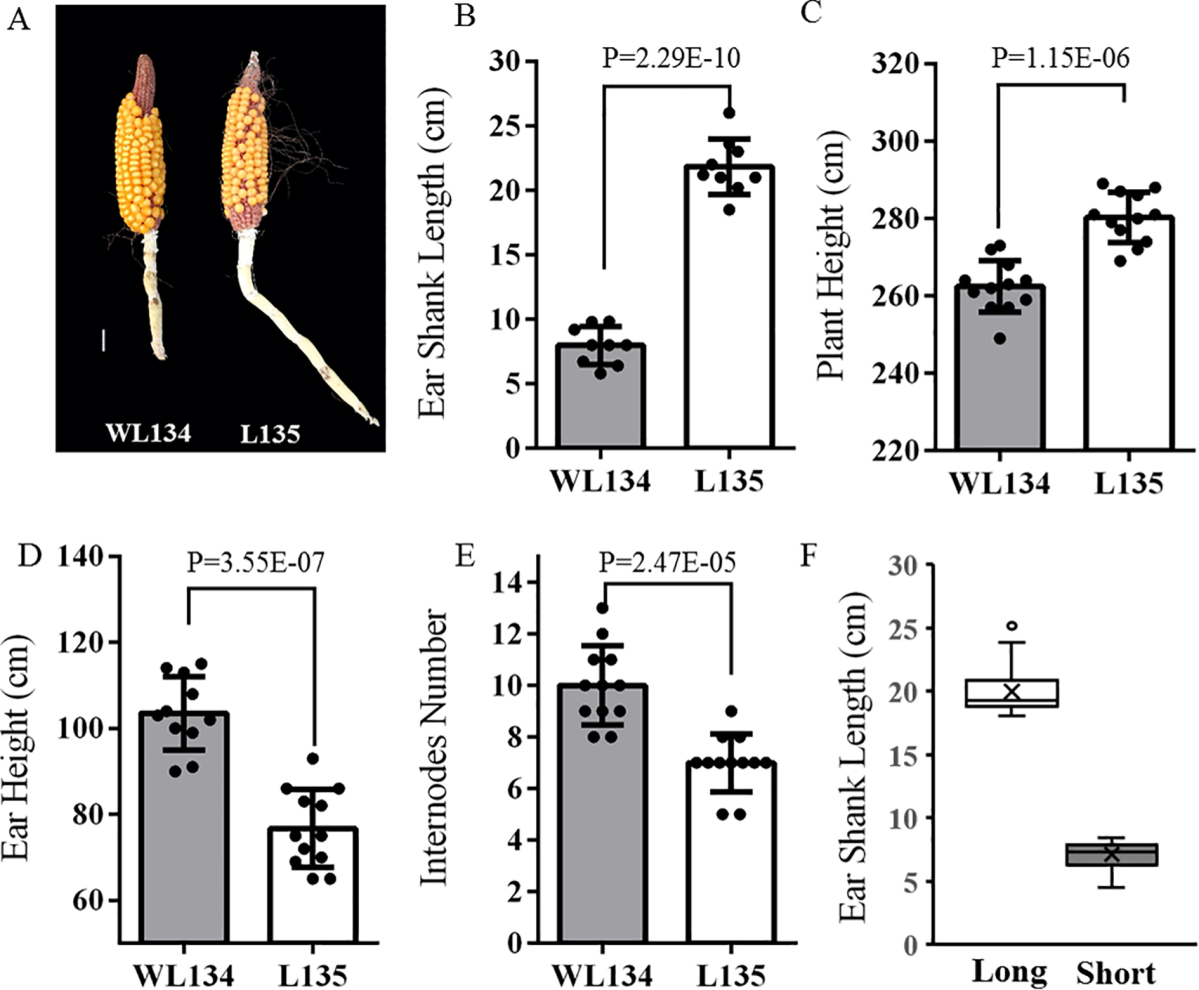
Phenotypes of ear shank length related traits in WL134 and L135. **(A)** Ear shank morphology of WL134 and L135, Scale bar = 2 cm. **(B)** Phenotypic of ear shank length. **(C, D)** Plant height and ear height phenotypes. **(E)** Number of internodes in the ear shank. **(F)** Ear shank length of extreme bulks.

Observing the phenotypic traits related to ear shank length in the two parental lines WL134 and L135, the average ear shank length of WL134 was 7.97 cm, while that of L135 was 21.83 cm. WL134 represents a relatively short ear shank material, with its ear shank length showing a significant difference compared to L135 (**Figures 1A, B**). Additionally, ear shank related traits including plant height, ear height and internodes number were examined in both parents. Significant differences were observed across these traits between WL134 and L135 (**Figures 1C-E**).

To map the genes regulating ear shank length, we constructed an F_2_ population with 308 lines derived from a cross between the long ear shank length inbred line L135 and the relatively short ear shank length inbred line WL134. This F_2_ population was used to identify phenotypic characterization of ear shank length. The F_2_ plants were phenotyped, 30 lines exhibiting long ear shank length (such as L135 and L1-30) and 30 individuals exhibiting short ear shank length (such as WL134 and S1-30) were selected based on ear shank length measurements to construct the corresponding extreme bulks (**Supplementary Figure S1**). The survey and analysis of ear shank length revealed a significant difference between the two extreme pools, which was suitable for follow-up research (**Figure 1F**). So, these selected lines were used to construct the extreme long ear shank length bulk and the extreme short ear shank length bulk for BSA-seq analysis.

### 3.2 Mapping of ear shank length genes based on BSA-seq in maize

Sequencing was performed on the parental materials as along with the extreme long ear shank bulk and short ear shank bulk derived from the F_2_ population (**Table 1**). A total of 31.59 million clean reads were generated for the relatively short ear shank parent WL134, 28.73 million clean reads were generated for the long ear shank parent L135. 40.47 million clean reads were generated for long ear shank bulk, and 45.48 million clean reads were generated for short ear shank bulk. The sequencing depths for WL134, L135, long ear shank bulk and short ear shank bulk were 22.63×, 20.47×, 25.51× and 30.38×, respectively. The properly paired ratios were more than 87.51% (97.40% for WL134, 96.01% for L135, 87.51% for L-pool, 93.13% for S-pool). All samples exhibited high data alignment rates, providing a reliable foundation for subsequent SNP detection.

**Table 1:** Coverage of the reads mapping to the B73 reference genome from the re-sequencing of WL134 and L135.

Sample Name	Clean reads	Mapped reads	Mapping rate(%)	Average depth(X)	Coverage 1X(%)	Coverage 4X(%)
WL134	315,879,956	307,680,179	97.4	22.63	90.92	84.5
L135	287,290,812	275,840,415	96.01	20.47	90.02	82.71
L_pool	404,687,854	354,128,281	87.51	25.51	92.64	87
S_pool	454,781,966	423,550,032	93.13	30.38	93.1	87.95

**Figure 2:**
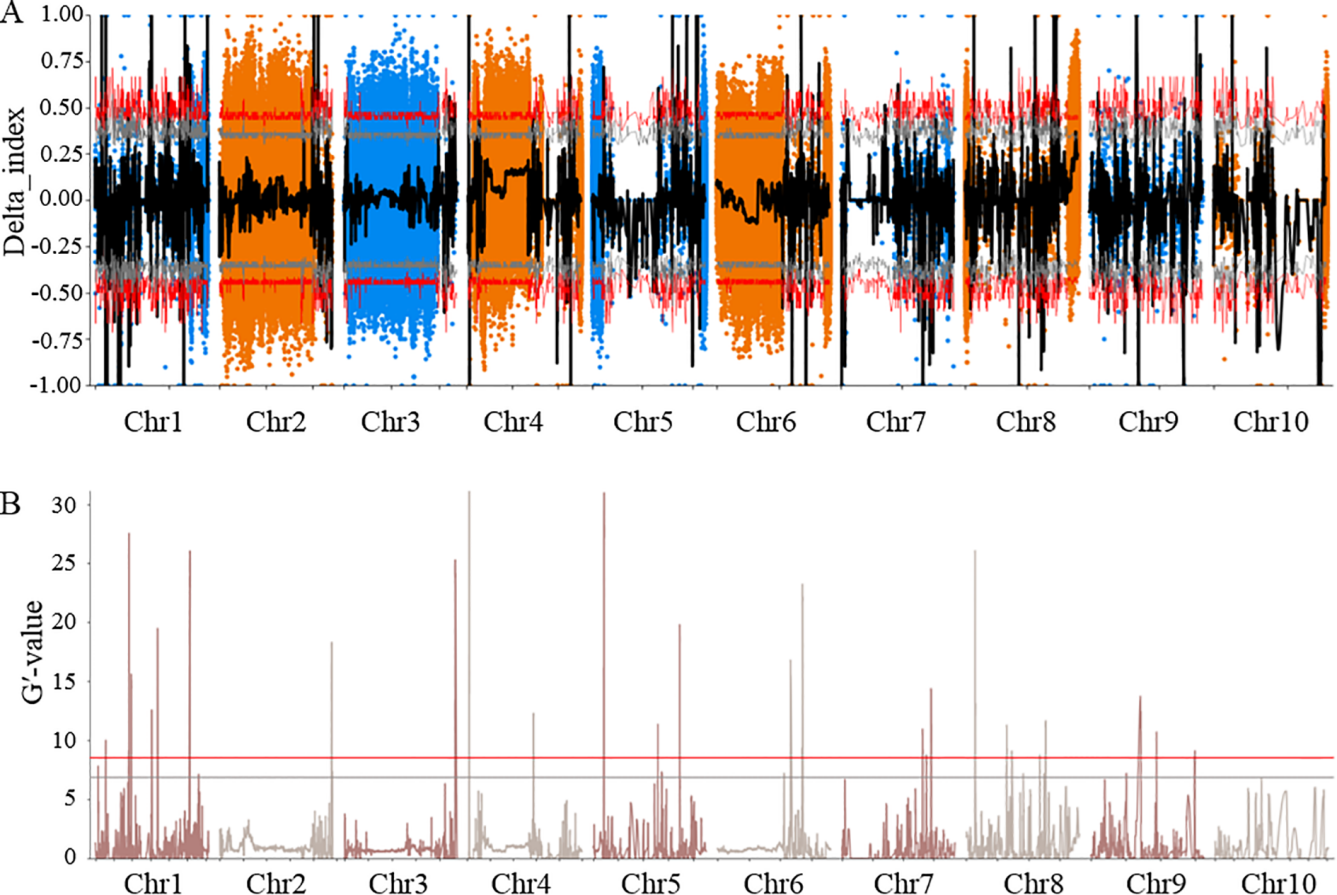
BSA: **(A)** △SNP/Indel-index method and **(B)** G′-value method. Red arrows indicate the top 1% threshold value in the two different methods.

A total of 1,503,803 SNPs and 197,389 Indels were finally detected by comparison and filtering between the two bulks (**Supplementary Figure S2**). Association analysis between the ear shank length trait and polymorphic markers were performed using the Δ(SNP/InDel-index) and G′-value methods. After overlapping the results obtained based on the 99% significance level (top 1%, red line) thresholds of the Δ(SNP/InDel-index) confidence intervals (**Figure 2A**) and the G′-value thresholds (**Figure 2B**), fourteen QTLs were identified (**Supplementary Table S2**). These loci were located on Chr1, Chr2, Chr4, Chr5, Chr6, Chr7, Chr8 and Chr9. Among them, *qESL1*, *qESL4*, *qESL5*, *qESL7* and *qESL10*, five loci co-localized with previously reported QTLs, whereas the remaining nine represent novel genomic regions associated with ear shank length.

### 3.3 RNA-seq analysis of ear shank length in the two parental lines

**Figure 3:**
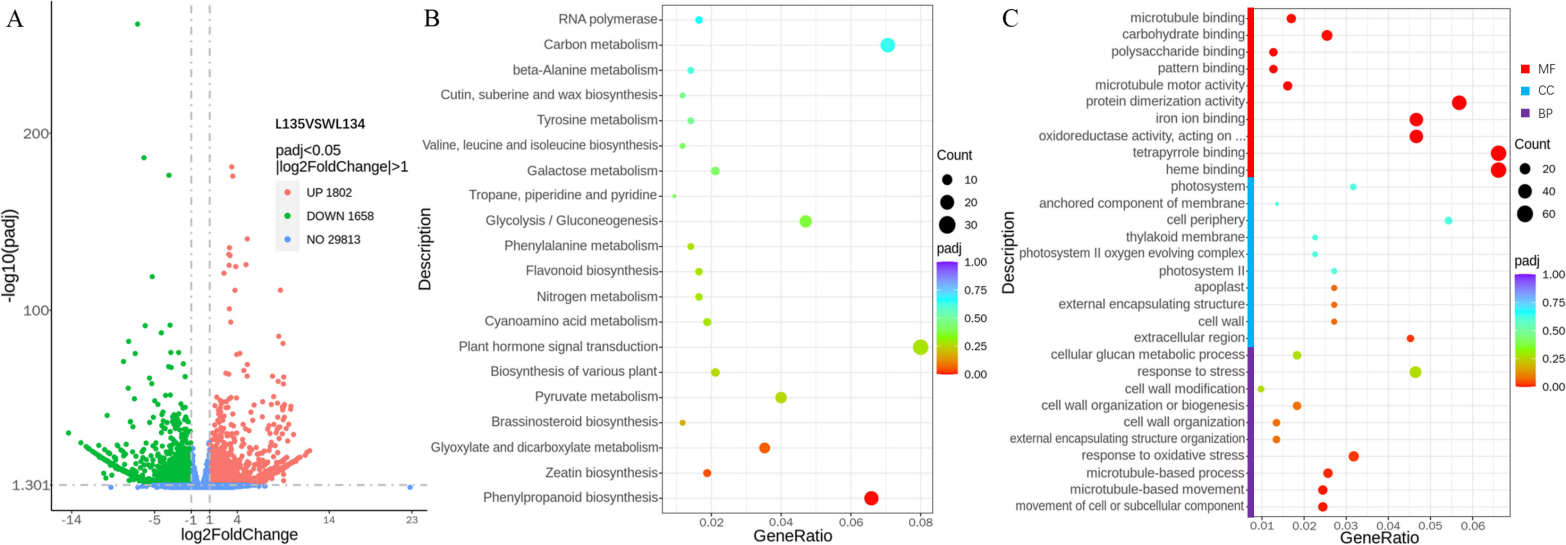
Differentially expressed genes (DEGs) between WL134 and L135. **(A)** Number of DEGs identified between the two inbred lines. **(B)** KEGG enrichment in two inbred lines. **(C)** Gene Ontology (GO) enrichment in two inbred lines.

To investigate the DEGs between the long ear shank line L135 and the relatively short ear shank line WL134, we performed transcriptome sequencing analysis on different ear shank samples at the silking stage. The RNA sequencing of six cDNA libraries (L135-1, L135-2, L135-3, WL134_1, WL134_2, WL134_3) were generated after filtering a total of 44.87 Gb clean bases (**Table 2**). The average percentages of Q20 and Q30 were 98.0% and 94.5%, respectively. 91.96% to 92.66% of the clean reads were successfully mapped to the reference genome B73_V5 using the HISAT2 software. Pearson correlation analysis revealed that the correlation between replicates was stronger than that different samples, further demonstrating the accuracy and reproducibility of the sequencing results (**Supplementary Figure S3**). A total of 3,460 DEGs were identified between the long ear shank and the short materials. Compared to the short ear shank material WL134, 1,802 genes were upregulated and 1,658 genes were downregulated in the long ear shank material L135 (**Figure 3A**).

**Table 2:** Summary of mapping reads and RNA-seq.

Sample	Raw base (bp)	Clean base (bp)	Effective rate (%)	Q20 (%)	Q30 (%)	Total_map (%)
L135_1	8.13G	8.01G	0.99	97.88	94.15	92.23
L135_2	6.26G	6.02G	0.96	97.95	94.32	92.12
L135_3	6.82G	6.36G	0.93	98.08	94.64	91.96
WL134_1	7.47G	7.17G	0.96	98.17	94.81	92.66
WL134_2	10.18G	9.86G	0.97	97.98	94.37	92.28
WL134_3	7.77G	7.45G	0.96	97.98	94.38	92.52

KEGG enrichment analysis revealed that all the DEGs were enriched in 113 metabolic pathways. The top 20 KEGG pathways were based on enrichment factor (**Figure 3B**). The DEGs were primarily involved in pathways such as phenylpropanoid biosynthesis, zeatin biosynthesis, glyoxylate and dicarboxylate metabolism, brassinosteroid biosynthesis and pyruvate metabolism, etc. The pathways including flavonoid biosynthesis, phenylalanine metabolism, tropane, piperidine and pyridine alkaloid biosynthesis and tyrosine metabolism were exclusively enriched among upregulated genes, while the remaining pathways were contained both upregulated and downregulated genes.

GO analysis was conducted to explore the BP, CC and MF associated with ear shank length variation between WL134 and L135. The top 10 GO terms were selected based on the number of enriched genes (**Figure 3C**). Common GO terms included movement of cell or subcellular component, microtubule-based movement, response to oxidative stress, external encapsulating structure organization and cell wall organization were enriched in BP. GO terms included extracellular region, cell wall, external encapsulating structure, apoplast and photosystem II were significantly enriched in CC. GO terms included heme binding, tetrapyrrole binding, incorporation or reduction of molecular oxygen, iron ion binding and protein dimerization activity were notable enriched in MF.

### 3.4 Combined analysis of BSA-seq and RNA-seq

**Figure 4:**
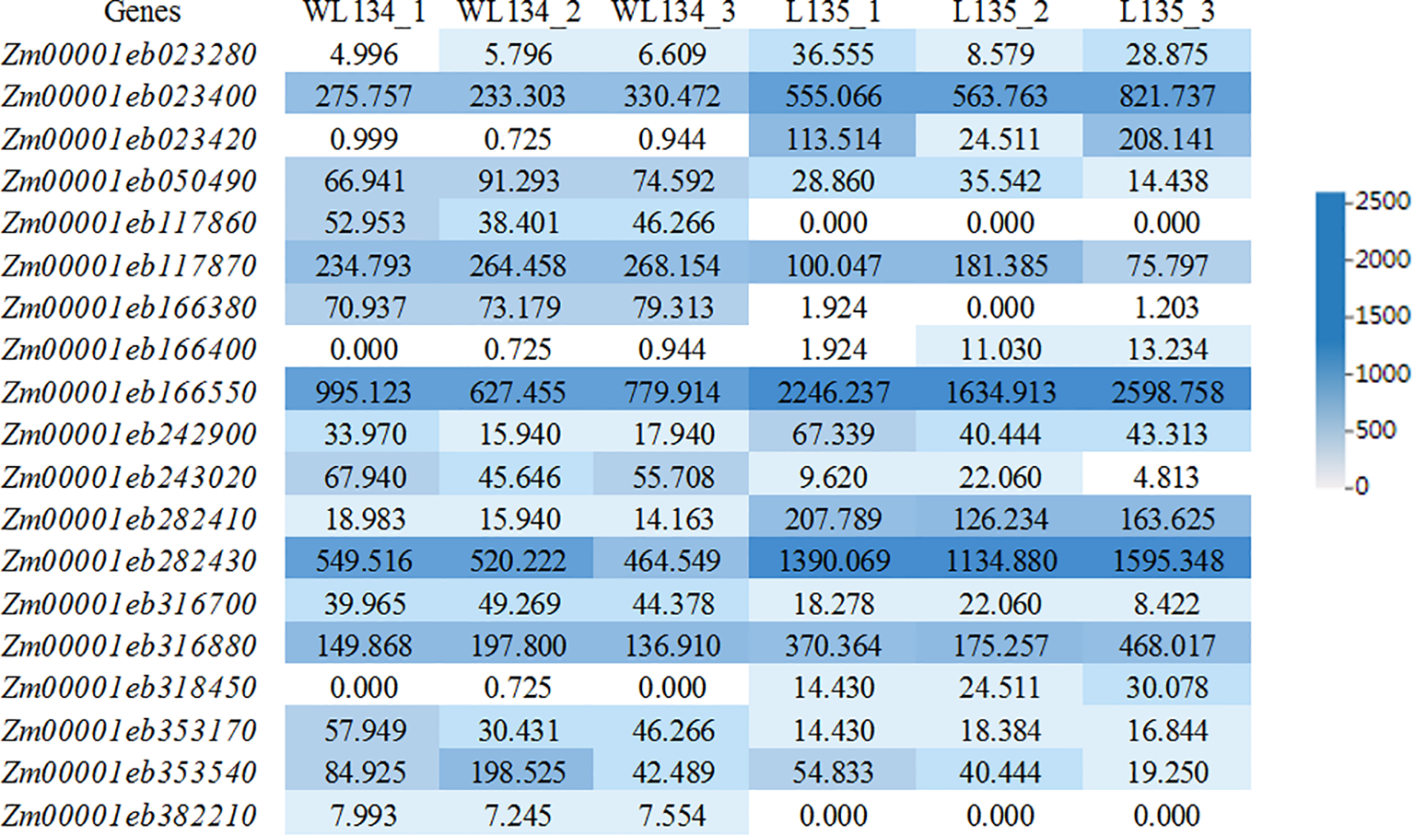
Expression analysis of nineteen genes by RNA-seq.

To further explore candidate genes associated with maize ear shank length, the results obtained by BSA-seq and RNA-seq were combined to analyze. Based on polymorphisms identified between the parents, we detected 334 genes containing nonsynonymous mutations, synonymous mutations and frameshift mutations in their open reading frames (ORFs) regions by BSA-seq. We compared the 334 genes obtained by BSA-seq with the 3460 DEGs obtained by RNA-seq, only nineteen common genes were selected. Through annotation analysis of SNPs within genes (**Supplementary Table S3**), it was found that the *Zm00001eb166380* gene had a frameshift insertion, while the *Zm00001eb023420*, *Zm00001eb166550* and *Zm00001eb353540* genes had frameshift deletions. Stopgain SNVs were present in the *Zm00001eb353540* and *Zm00001eb382210* genes. Synonymous SNVs, nonsynonymous SNVs, nonframeshift deletions or nonframeshift insertions were present in the exons of the other genes. The RNA-seq results revealed differential expression of these 19 genes between the two parental lines (**Figure 4**). Among these, five genes (*Zm00001eb023280*, *Zm00001eb282410*, *Zm00001eb282430*, *Zm00001eb353170* and *Zm00001eb353540*) were enriched in BP ontologies. One gene (*Zm00001eb023280*) was enriched in CC ontologies, and six genes (*Zm00001eb023280*, *Zm00001eb166400*, *Zm00001eb282410*, *Zm00001eb282430*, *Zm00001eb316880* and *Zm00001eb353540*) were enriched in MF ontologies by GO function (**Supplementary Table S3**).

### 3.5 Validation of candidate genes by qRT-PCR

**Figure 5:**
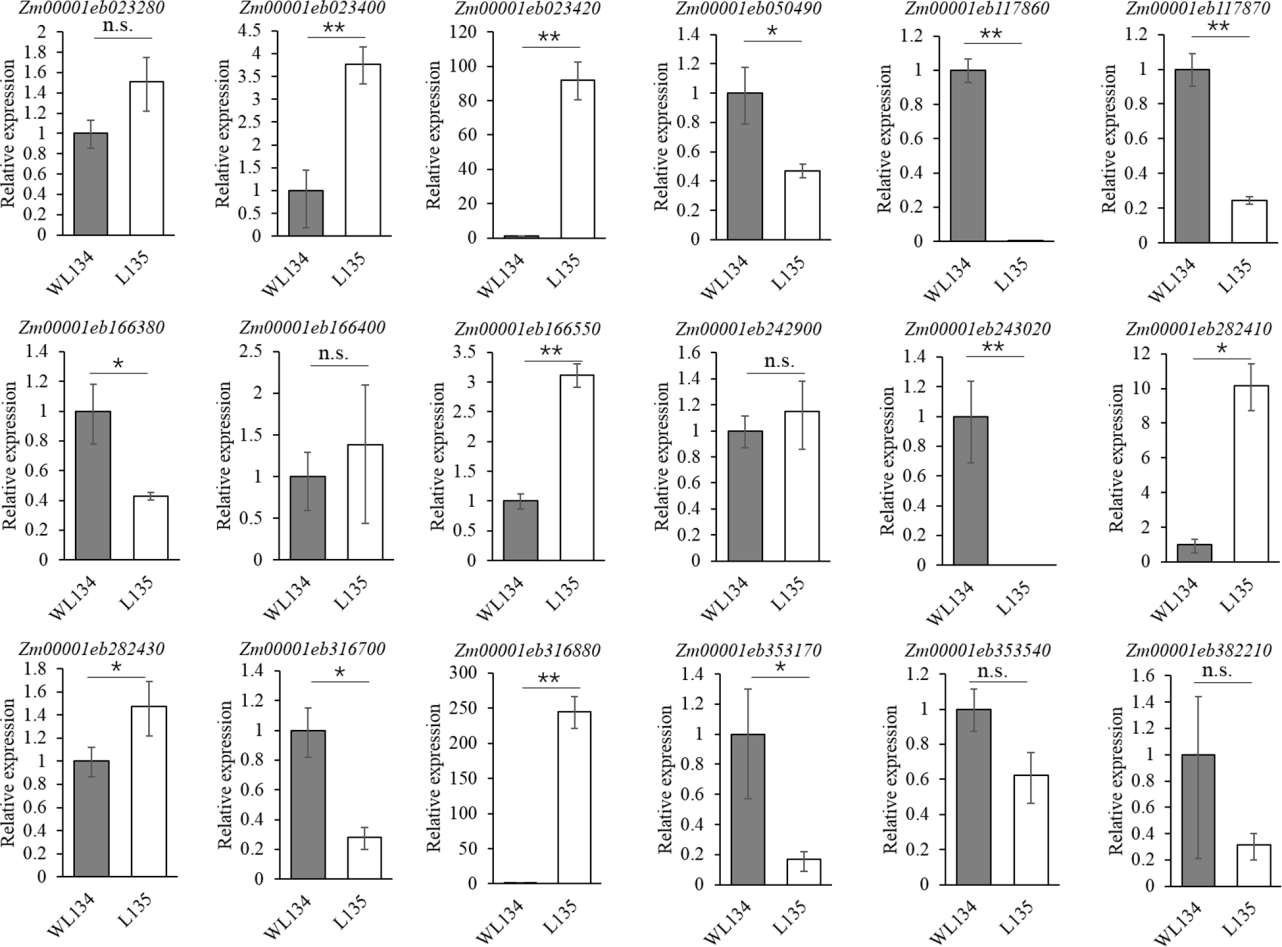
qRT-PCR confirmation of the differentially expressed genes. *, p < 0.05; **, p < 0.01.

To further confirm whether the candidate genes exhibit differential expression levels between the two parental lines and to validate the accuracy of the transcriptomic differential expression data, associated DEGs identified through the integrated BSA-seq and RNA-seq analysis were selected for validation using quantitative real-time PCR (qRT-PCR). These results indicated that the expression trends of these genes aligned consistently with the RNA-seq results (**Figure 5**). Among them, six genes including *Zm00001eb023400*, *Zm00001eb023420*, Zm00001eb166550, Zm00001eb316880, *Zm00001eb282410* and *Zm00001eb282430* were significantly upregulated in the long ear shank lines. Conversely, seven genes including *Zm00001eb117860*, *Zm00001eb117870*, *Zm00001eb243020*, *Zm00001eb050490*, *Zm00001eb166380*, *Zm00001eb316700* and *Zm00001eb353170* were significantly downregulated (**Table 3**). Consequently, the integration of BSA-seq and RNA-seq analyses identified 13 genes as high-confidence candidates within the targeted genomic regions.

**Table 3:** Annotation information of 13 candidate genes.

Genes	DEG type	Chr	Location	Annotation
Zm00001eb023400	Up	1	92565596	Lytic transglycolase
Zm00001eb023420	Up	1	92660743	Lytic transglycolase
Zm00001eb050490	Down	1	257309316	Lytic transglycolase
Zm00001eb117860	Down	2	241768084	ATPase family AAA domain-containing protein 3
Zm00001eb117870	Down	2	241771983	ATP-dependent DNA helicase DDM1
Zm00001eb166380	Down	4	5753412	RING domain ligase 2
Zm00001eb166550	Up	4	6007762	Glycine-rich domain-containing protein-like
Zm00001eb243020	Down	5	173207819	Protein SMAX1-LIKE 3
Zm00001eb282410	Up	6	135796741	Plasma membrane-bound peroxidase 1
Zm00001eb282430	Up	6	135946028	Peroxidase
Zm00001eb316700	Down	7	138417429	IRK-interacting protein
Zm00001eb316880	Up	7	138936978	Wall-associated receptor kinase
Zm00001eb353170	Down	8	126297959	non-specific serine/threonine protein kinase

*Zm00001eb023400* and *Zm00001eb023420* on chromosome 1 exhibited upregulation, whereas *Zm00001eb050490* showed downregulation in comparison to the short ear shank length line WL134 (**Table 3**). All three genes encoded lytic transglycolase situated in the membrane, primarily functioning as components of the cell wall or cellular component curator. On chromosome 2, *Zm00001eb117860* and *Zm00001eb117870* were downregulated. The two genes belonged to ATPase family and regulated the binding of sequence specific DNA. Based on the annotation information, *Zm00001eb166380* and *Zm00001eb166550* on chromosome 4 encoded ring domain ligase 2 and glycine rich domain containing protein respectively. *Zm00001eb243020* on chromosome 5 encoded protein smax1-like 3. On chromosome 6, *Zm00001eb282410* and *Zm00001eb282430* encoded peroxidase, all of which were enriched in phenylpropanoid biosynthesis pathway. *Zm00001eb316700* and *Zm00001eb316880* on chromosome 7 encode IRK-interacting protein and wall associated receptor kinase respectively. *Zm00001eb353170* on chromosome 8 encoded non-specific serine/threonine protein kinase.

## 4 Discussion

The maize ear shank is a lateral stalk connecting the ear to the main stalk, transporting photosynthetic products from the leaves to the kernels. As the sole channel for directing assimilates from the vegetative tissues to the ear, the traits of the ear shank significantly influence grain yield. Selecting appropriate ear shank length in breeding is not only beneficial for grain yield accumulation but also ear dehydration and mechanical harvesting. Therefore, identifying QTLs associated with ear shank length and predicting related candidate genes can provide a theoretical foundation for molecular improvement of this trait. In our study, the ear shank length of L135 was approximately three times that of WL134, indicating that these two inbred lines can serve as ideal materials for investigating the regulatory genes of ear shank length in maize.

In this study, BSA-seq analysis using the Δ(SNP/InDel-index) and G′-value methods identified 14 QTL loci. Comparing these with previously reported QTLs associated with maize ear shank length traits, five loci were found to be consistent with prior findings. The *qESL1* and *qESL5* identified on chromosomes 1 and 4 in this study exhibited close to or partially overlapping with the *qSL BYD-1–1* and *qSL BYK-4–1* in physical positions (Liu M et al., 2021), suggesting potential regulation by the same genetic locus. Similarly, the interval covered by *qESL4* and *qESL7* mapped on chromosomes 2 and 5 in this study was found to coincide with that of the *qESL2–3* and *qESL5–2* previously detected (Liang et al., 2022). Additionally, the *qESL10* identified on chromosome 7 in this study aligned closely with the *qPVB43* (Sun et al., 2022), further supporting its potential functional relevance. The consistency between these findings and earlier studies reinforces the reliability and accuracy of the results obtained in this research.

This study combines BSA-seq and RNA-seq to identify QTL loci, SNPs and DEGs associated with maize ear shank elongation. Similar strategies have been widely applied to uncover key regulatory genes for various traits in plants. The method of BSA-seq combined with RNA-seq was used to identify genes associated with plant height in foxtail millet (Gao et al., 2022). New genes modulating salt tolerance in maize were identified using the combination of RNA-seq and BSA-seq (Zhu et al., 2023). Additionally, the combination of RNA-seq and BSA-seq was utilized to identify candidate genes regulating seed storability in wild rice (Zhou et al., 2025). The integration of BSA-seq and RNA-seq approaches led to the identification of candidate genes associated with seed weight in *Brassica napus* (Geng et al., 2025). The overlapping display genes of BSA seq and DEGs were validated using qRT-PCR. The results were consistent with the gene expression trend in RNA-seq, indicating the reliability of transcriptome data.

Functional analysis was conducted on three candidate genes, *Zm00001eb023400*, *Zm00001eb023420* and *Zm00001eb050490*, encoding lytic transglycosylases classified in glycoside hydrolase family 45 (GH45). These enzymes were involved in cell wall modification, encompassing remodeling and degradation (Bharadwaj et al., 2020). The elongation of stalk internodes depended on the elongation of longitudinal cells or an increase in cell number through cell division (Zhang et al., 2020). Cell division was often accompanied by irreversible changes to the cell wall, which was composed of a network of polysaccharides including cellulose and xylan (Liu C et al., 2021). The ear shank belongs to lateral stalk, and its length regulation might be related to the regulation of internode elongation involving glycoside hydrolase.

As a core pathway in plant secondary metabolism, phenylpropanoid biosynthesis produced a variety of compounds that played crucial roles in regulating plant height and cell elongation (Dong and Lin, 2021). Lignin, a key product of this pathway, significantly influenced plant height by modulating cell wall development (Fernández-Pérez et al., 2015). Overexpression of *ZmMYB69* in maize had been shown to decrease lignin content in the cell wall, leading to plant dwarfing and vascular bundle cell wall thinning (Qiang et al., 2022). Flavonoids, another products derived from phenylpropanoid biosynthesis pathway, had been proposed as endogenous auxin transport inhibitors in plants. These synthetic auxin transport inhibitors influence the auxin signaling pathway, thereby affecting cell elongation and differentiation (Brown et al., 2001). *Zm00001eb282410* and *Zm00001eb282430* identified in this study were enriched in the phenylpropanoid biosynthesis pathway. They might play a role in regulating panicle stem length. Furthermore, genes encoding ATPase, receptor kinase and serine/threonine protein kinase exhibited significant differential expression between long and short ear shanks. Consequently, we identified 13 DEGs involved in the regulation of ear shank length, which might serve as important candidate genes associated with maize ear shank elongation.

## 5 Conclusion

In this study, BSA-seq and RNA-seq combined analysis was constructed by resequencing to identify QTLs and candidate genes related to ear shank elongation in maize. Fourteen loci distributed on chromosomes 1, 2, 4, 5, 6, 7, 8 and 9 were identified and thirteen candidate genes were selected in the targeted regions. These genes are mainly involved in cell wall removal and degradation, as well as phenylpropanoid biosynthesis pathway, including *Zm00001eb023400*, *Zm00001eb023420*, *Zm00001eb050490*, *Zm00001eb282410* and *Zm00001eb282430* for regulating ear shank length. This study provides a molecular basis for the genetic improvement of ear shank length and an important resource for the breeding of high-density varieties in maize. Functional validation is needed to confirm the biological functions of these genes in the future.

## Data Availability

The datasets presented in this study are available in NCBI, Accession number:PRJNA1414085 and PRJNA1414693.
