# Supplementary material for: Identification of QTLs and new candidate genes affecting ear shank length via BSA-seq and transcriptomic analysis in maize
Source: Front Plant Sci. 2026 Feb 9;17:1768852. doi: 10.3389/fpls.2026.1768852 (PMC12926374; doi:10.3389/fpls.2026.1768852)
Supplement: Supplementary file 1 [file DataSheet1.doc]

Supplementary Material

# Supplementary Figures and Tables

## Supplementary Figures

##
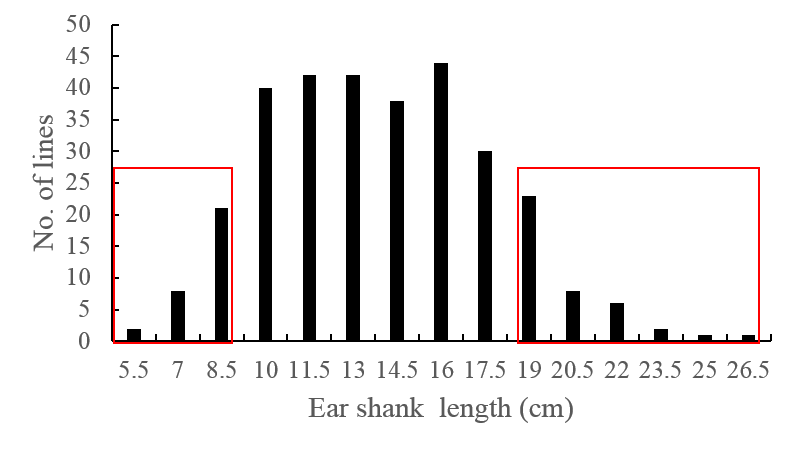


## Supplementary Figure 1. Frequency distribution of ear shank length in the WL134/L135 F_2_ population.

##
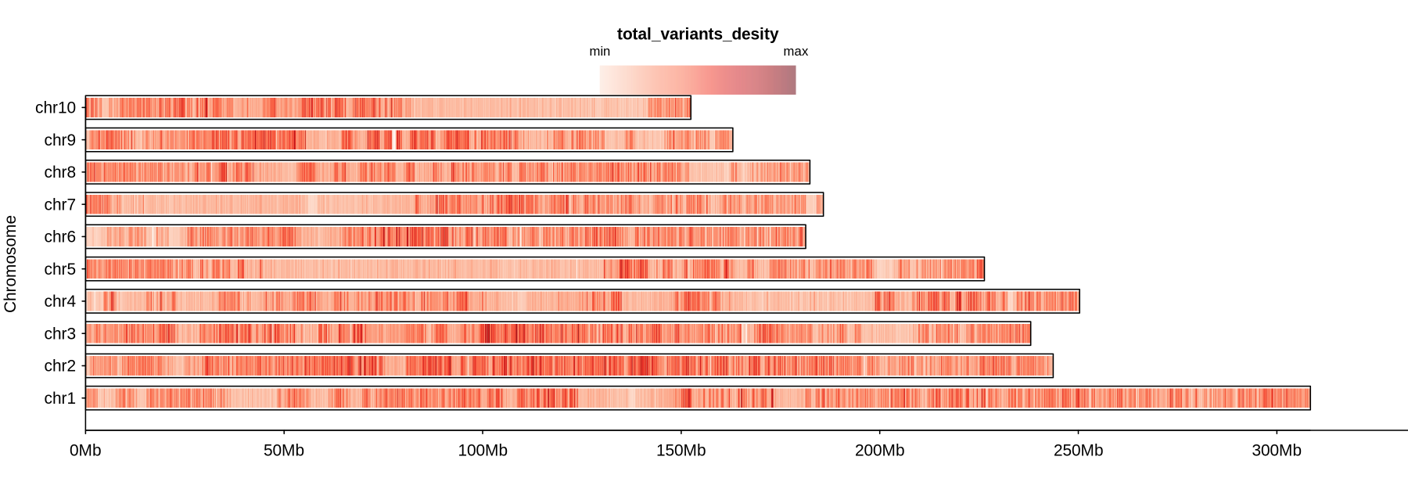
Supplementary Figure 2. The distribution of total variations (1,503,803 SNPs and 197,389 Indels) in chromosomes.

**
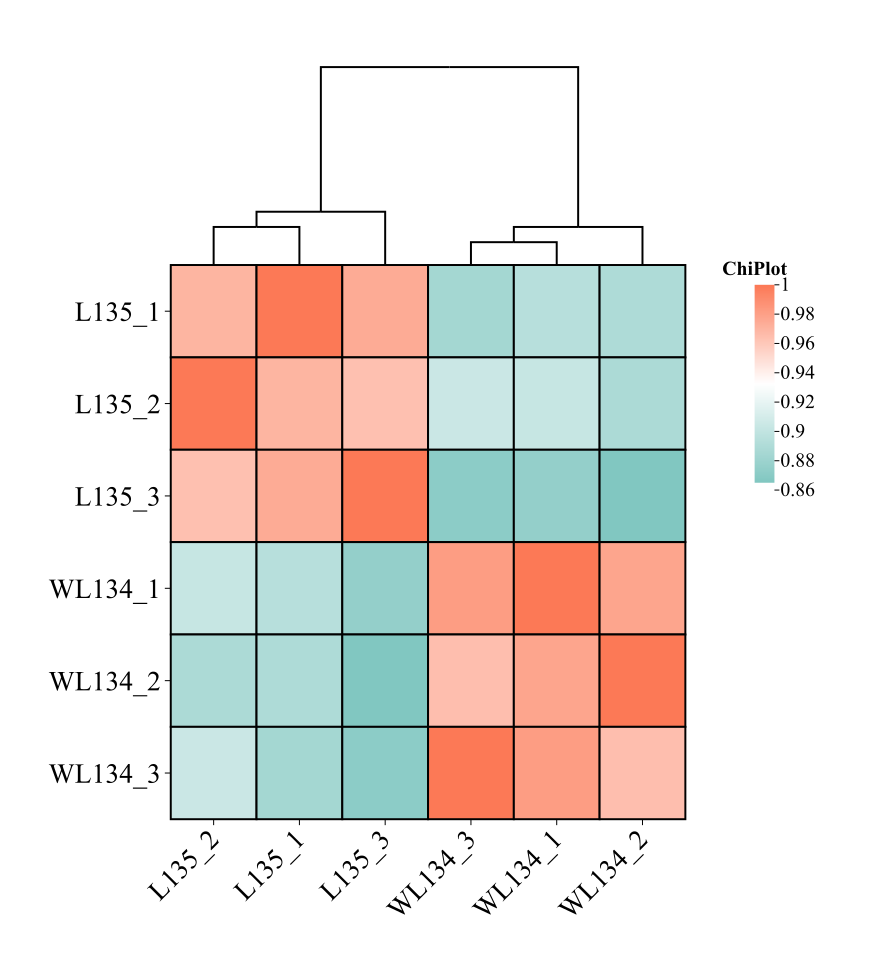
**

**Supplementary Figure 3.** The Pearson correlation analysis between six samples.

## Supplementary Tables

**Table S1** The list of primer for qPCR.

| **Name** | **Sequence** |
| --- | --- |
| Zm00001eb023280-F | GACGCACTTGTAGATAGA |
| Zm00001eb023280-R | TTACTGTCCAAATACCAAAC |
| Zm00001eb023400-F | CCGCTCCATCATTCAATA |
| Zm00001eb023400-R | GATACAATTACATACAGTTCCAA |
| Zm00001eb023420-F | TCTATCGTCCAGTATTAGC |
| Zm00001eb023420-R | AGCACAATGACACAGAAT |
| Zm00001eb050490-F | GCTGCTGCTGGAACATTT |
| Zm00001eb050490-R | CATCATAATTCACAACGCCAATAC |
| Zm00001eb117860-F | GCTCCTTTCTTCCTCCTC |
| Zm00001eb117860-R | CGTAGAAACCCAGTCACC |
| Zm00001eb117870-F | AATCTTGTGCCGTTATTG |
| Zm00001eb117870-R | ATCAGCCAGTATTCCATT |
| Zm00001eb166380-F | GGTTACGAGGAGTGTAGATA |
| Zm00001eb166380-R | CCTTCACAATGGCATCAA |
| Zm00001eb166400-F | TCGCCTTAGTTGTCATTC |
| Zm00001eb166400-R | GTTCCTGGTATTATCTCTGTAA |
| Zm00001eb166550-F | TTGGTTGGAGTATTGAAGTCA |
| Zm00001eb166550-R | TTATTGGCAAGTTGGAGTCT |
| Zm00001eb242900-F | CCGAGGAAGTACAAGAGT |
| Zm00001eb242900-R | GAAGAGATAGGTTGAGTTTACAT |
| Zm00001eb243020-F | CTGATGAGAGTAGCGATTA |
| Zm00001eb243020-R | TCCATTCTTCCAACACAA |
| Zm00001eb282410-F | GATGTCTAGTGTGAGTTG |
| Zm00001eb282410-R | TTCAATAATCCGATGAGTATA |
| Zm00001eb282430-F | AAGAAGAAGCAGGTGATG |
| Zm00001eb282430-R | ACTAATAATCAGATGTGGTCATA |
| Zm00001eb316700-F | GCTCATTATCTGATCTGTC |
| Zm00001eb316700-R | TTACCACACCAATATCCA |
| Zm00001eb316880-F‘ | AGACAGTGCTTAGAGATGA |
| Zm00001eb316880-R | TCTTCCTCAACCTACCAA |
| Zm00001eb318450-F | TGAAGGCAATAAGATAGG |
| Zm00001eb318450-R | ACAAGAACGGATACATAA |
| Zm00001eb353170-F | GCTTAGAAGGTAACTCTTG |
| Zm00001eb353170-R | TATTAGCACACGACTACT |
| Zm00001eb353540-F | GTAGCACGCCACATTGTT |
| Zm00001eb353540-R | GCTCTTCATTCACATCAACTAAT |
| Zm00001eb382210-F | AGGCTAACAACACCATCAA |
| Zm00001eb382210-R | CTCTCCACTTCTCTTCTTCTT |
| Tubulin-F | CCGATCTCACACATTTTGGAAA |
| Tubulin-R | GATGATCTCTCTCATCCTGCTC |

**Table S2** QTLs were detected by △SNP/Indel-index and G-value method with the top 1% threshold value.

| **QTLs** | **Chr** | **Methods** | **Start** | **End** | **Length** | **Gene_num** | **Known loci** |
| --- | --- | --- | --- | --- | --- | --- | --- |
| *qESL1* | chr1 | G-value | 28100000 | 29300000 | 1200000 | 33 | *qSL BYD-1-1*, Liu etal, 2021 |
|  | chr1 | △SNP/Indel-index | 28800000 | 30200000 | 1400000 | 29 |  |
| *qESL2* | chr1 | G-value | 90800000 | 92700000 | 1900000 | 25 |  |
|  | chr1 | △SNP/Indel-index | 91400000 | 92700000 | 1300000 | 19 |  |
| *qESL3* | chr1 | G-value | 256500000 | 257800000 | 1300000 | 28 |  |
|  | chr1 | △SNP/Indel-index | 256500000 | 257800000 | 1300000 | 28 |  |
| *qESL4* | chr2 | G-value | 239100000 | 242000000 | 2900000 | 136 | *qESL2-3* , Liang et al., 2022 |
|  | chr2 | △SNP/Indel-index | 239300000 | 240400000 | 1100000 | 48 |  |
|  | chr2 | △SNP/Indel-index | 241000000 | 242100000 | 1100000 | 45 |  |
| *qESL5* | chr4 | G-value | 4500000 | 6400000 | 1900000 | 98 | *qSL BYK-4-1*, Liu etal, 2021 |
|  | chr4 | △SNP/Indel-index | 5100000 | 7900000 | 2800000 | 127 |  |
| *qESL6* | chr5 | G-value | 136700000 | 138100000 | 1400000 | 12 |  |
|  | chr5 | △SNP/Indel-index | 136700000 | 138100000 | 1400000 | 12 |  |
| *qESL7* | chr5 | G-value | 172300000 | 173500000 | 1200000 | 28 | *qESL5-2* , Liang et al., 2022 |
|  | chr5 | △SNP/Indel-index | 172300000 | 173500000 | 1200000 | 28 |  |
| *qESL8* | chr6 | G-value | 134700000 | 136600000 | 1900000 | 43 |  |
|  | chr6 | △SNP/Indel-index | 135400000 | 136600000 | 1200000 | 29 |  |
| *qESL9* | chr7 | G-value | 138300000 | 139600000 | 1300000 | 43 |  |
|  | chr7 | △SNP/Indel-index | 138300000 | 139600000 | 1300000 | 43 |  |
| *qESL10* | chr7 | G-value | 145400000 | 147300000 | 1900000 | 46 | *qPVB43* , Sun et al., 2022 |
|  | chr7 | △SNP/Indel-index | 145400000 | 147300000 | 1900000 | 46 |  |
| *qESL11* | chr8 | G-value | 14200000 | 15700000 | 1500000 | 40 |  |
|  | chr8 | △SNP/Indel-index | 13700000 | 15000000 | 1300000 | 32 |  |
| *qESL12* | chr8 | G-value | 124700000 | 128400000 | 3700000 | 101 |  |
|  | chr8 | △SNP/Indel-index | 125100000 | 126400000 | 1300000 | 35 |  |
|  | chr8 | △SNP/Indel-index | 127000000 | 128100000 | 1100000 | 33 |  |
| *qESL13* | chr9 | G-value | 50900000 | 52100000 | 1200000 | 12 |  |
|  | chr9 | △SNP/Indel-index | 50300000 | 51600000 | 1300000 | 16 |  |
| *qESL14* | chr9 | G-value | 70800000 | 72700000 | 1900000 | 24 |  |
|  | chr9 | △SNP/Indel-index | 70200000 | 71700000 | 1500000 | 17 |  |

**Table S3 SNP annotation and GO analysis of 19 genes.**

| **CHR** | **POS** | **Exon_varSNP** | **Variant** | **Gene_name** | **REF** | **ALT** | **Type** | **GO** |
| --- | --- | --- | --- | --- | --- | --- | --- | --- |
| chr1 | 91893198 | synonymous SNV | exonic | *Zm00001eb023280* | G | T | snp | BP; CC; MF |
| chr1 | 91893627 | synonymous SNV | exonic |  | T | C | snp |  |
| chr1 | 91893738 | synonymous SNV | exonic |  | A | G | snp |  |
| chr1 | 91893942 | synonymous SNV | exonic |  | T | C | snp |  |
| chr1 | 92565668 | nonframeshift deletion | exonic | *Zm00001eb023400* | CCTT | C | InDel | NA |
| chr1 | 92565825 | synonymous SNV | exonic |  | T | C | snp |  |
| chr1 | 92566035 | synonymous SNV | exonic |  | C | A | snp |  |
| chr1 | 92566310 | synonymous SNV | exonic |  | T | G | snp |  |
| chr1 | 92566313 | synonymous SNV | exonic |  | T | C | snp |  |
| chr1 | 92566334 | synonymous SNV | exonic |  | A | C | snp |  |
| chr1 | 92566343 | nonsynonymous SNV | exonic |  | C | G | snp |  |
| chr1 | 92566548 | synonymous SNV | exonic |  | A | G | snp |  |
| chr1 | 92566869 | synonymous SNV | exonic |  | A | G | snp |  |
| chr1 | 92660943 | nonframeshift deletion | exonic | *Zm00001eb023420* | AGCT | A | InDel | NA |
| chr1 | 92660947 | frameshift deletion | exonic |  | AG | A | InDel |  |
| chr1 | 92660980 | nonframeshift deletion | exonic |  | CACA | C | InDel |  |
| chr1 | 92661061 | synonymous SNV | exonic |  | G | A | snp |  |
| chr1 | 92661151 | synonymous SNV | exonic |  | C | T | snp |  |
| chr1 | 92661294 | synonymous SNV | exonic |  | T | C | snp |  |
| chr1 | 92661312 | synonymous SNV | exonic |  | A | G | snp |  |
| chr1 | 92661480 | synonymous SNV | exonic |  | G | T | snp |  |
| chr1 | 257311360 | synonymous SNV | exonic | *Zm00001eb050490* | A | C | snp | NA |
| chr1 | 257311483 | synonymous SNV | exonic |  | C | T | snp |  |
| chr1 | 257311861 | synonymous SNV | exonic |  | T | A | snp |  |
| chr1 | 257311960 | synonymous SNV | exonic |  | A | G | snp |  |
| chr1 | 257311987 | synonymous SNV | exonic |  | C | T | snp |  |
| chr1 | 257312184 | synonymous SNV | exonic |  | T | C | snp |  |
| chr1 | 257312454 | synonymous SNV | exonic |  | A | G | snp |  |
| chr1 | 257312468 | nonsynonymous SNV | exonic |  | T | C | snp |  |
| chr1 | 257312599 | synonymous SNV | exonic |  | T | C | snp |  |
| chr2 | 241768221 | nonsynonymous SNV | exonic | *Zm00001eb117860* | A | G | snp | NA |
| chr2 | 241768227 | nonsynonymous SNV | exonic |  | G | A | snp |  |
| chr2 | 241768237 | synonymous SNV | exonic |  | A | T | snp |  |
| chr2 | 241768241 | nonsynonymous SNV | exonic |  | G | A | snp |  |
| chr2 | 241768251 | nonframeshift deletion | exonic |  | TTGC | T | InDel |  |
| chr2 | 241768252 | nonframeshift deletion | exonic |  | T | C | snp |  |
| chr2 | 241768274 | nonsynonymous SNV | exonic |  | G | T | snp |  |
| chr2 | 241768316 | nonsynonymous SNV | exonic |  | T | C | snp |  |
| chr2 | 241768326 | nonsynonymous SNV | exonic |  | A | C | snp |  |
| chr2 | 241768327 | synonymous SNV | exonic |  | G | A | snp |  |
| chr2 | 241768329 | nonsynonymous SNV | exonic |  | C | T | snp |  |
| chr2 | 241768351 | nonsynonymous SNV | exonic |  | C | A | snp |  |
| chr2 | 241768361 | nonsynonymous SNV | exonic |  | G | A | snp |  |
| chr2 | 241768368 | nonsynonymous SNV | exonic |  | C | T | snp |  |
| chr2 | 241768384 | synonymous SNV | exonic |  | G | A | snp |  |
| chr2 | 241768407 | nonsynonymous SNV | exonic |  | A | G | snp |  |
| chr2 | 241772989 | nonsynonymous SNV | exonic | *Zm00001eb117870* | C | A | snp | NA |
| chr2 | 241772991 | nonsynonymous SNV | exonic |  | A | C | snp |  |
| chr2 | 241773015 | nonsynonymous SNV | exonic |  | G | T | snp |  |
| chr2 | 241775597 | synonymous SNV | exonic |  | T | C | snp |  |
| chr2 | 241775998 | synonymous SNV | exonic |  | T | C | snp |  |
| chr2 | 241776052 | nonsynonymous SNV | exonic |  | G | T | snp |  |
| chr2 | 241776211 | synonymous SNV | exonic |  | C | T | snp |  |
| chr2 | 241776280 | synonymous SNV | exonic |  | G | A | snp |  |
| chr2 | 241776567 | synonymous SNV | exonic |  | C | T | snp |  |
| chr2 | 241776807 | synonymous SNV | exonic |  | C | T | snp |  |
| chr2 | 241776825 | synonymous SNV | exonic |  | C | G | snp |  |
| chr2 | 241776984 | nonsynonymous SNV | exonic |  | G | C | snp |  |
| chr2 | 241777334 | nonsynonymous SNV | exonic |  | T | C | snp |  |
| chr4 | 5754544 | nonsynonymous SNV | exonic | *Zm00001eb166380* | A | G | snp | NA |
| chr4 | 5754758 | synonymous SNV | exonic |  | T | C | snp |  |
| chr4 | 5755218 | synonymous SNV | exonic |  | G | A | snp |  |
| chr4 | 5755484 | nonsynonymous SNV | exonic |  | G | T | snp |  |
| chr4 | 5755644 | frameshift insertion | exonic |  | G | GGCGCTGTC | InDel |  |
| chr4 | 5755662 | synonymous SNV | exonic |  | T | C | snp |  |
| chr4 | 5755667 | nonsynonymous SNV | exonic |  | T | C | snp |  |
| chr4 | 5755673 | nonsynonymous SNV | exonic |  | C | T | snp |  |
| chr4 | 5755675 | nonsynonymous SNV | exonic |  | A | G | snp |  |
| chr4 | 5755736 | nonsynonymous SNV | exonic |  | T | C | snp |  |
| chr4 | 5755820 | nonsynonymous SNV | exonic |  | A | G | snp |  |
| chr4 | 5782421 | synonymous SNV | exonic | *Zm00001eb166400* | C | T | snp | MF |
| chr4 | 5782562 | nonsynonymous SNV | exonic |  | G | T | snp |  |
| chr4 | 5782614 | synonymous SNV | exonic |  | A | C | snp |  |
| chr4 | 5782653 | synonymous SNV | exonic |  | A | C | snp |  |
| chr4 | 6009684 | nonsynonymous SNV | exonic | *Zm00001eb166550* | C | A | snp | NA |
| chr4 | 6010148 | synonymous SNV | exonic |  | G | C | snp |  |
| chr4 | 6010235 | synonymous SNV | exonic |  | C | G | snp |  |
| chr4 | 6010687 | synonymous SNV | exonic |  | G | A | snp |  |
| chr4 | 6011148 | synonymous SNV | exonic |  | G | C | snp |  |
| chr4 | 6011239 | nonsynonymous SNV | exonic |  | G | T | snp |  |
| chr4 | 6011413 | nonsynonymous SNV | exonic |  | T | G | snp |  |
| chr4 | 6011427 | synonymous SNV | exonic |  | C | T | snp |  |
| chr4 | 6011780 | nonsynonymous SNV | exonic |  | G | A | snp |  |
| chr4 | 6011882 | nonsynonymous SNV | exonic |  | G | A | snp |  |
| chr4 | 6011914 | synonymous SNV | exonic |  | A | G | snp |  |
| chr4 | 6011974 | synonymous SNV | exonic |  | G | A | snp |  |
| chr4 | 6012035 | nonsynonymous SNV | exonic |  | G | A | snp |  |
| chr4 | 6012040 | synonymous SNV | exonic |  | A | C | snp |  |
| chr4 | 6012105 | nonsynonymous SNV | exonic |  | C | G | snp |  |
| chr4 | 6012114 | nonsynonymous SNV | exonic |  | G | A | snp |  |
| chr4 | 6012124 | synonymous SNV | exonic |  | A | G | snp |  |
| chr4 | 6012194 | nonsynonymous SNV | exonic |  | G | A | snp |  |
| chr4 | 6012522 | nonsynonymous SNV | exonic |  | A | G | snp |  |
| chr4 | 6012530 | synonymous SNV | exonic |  | C | A | snp |  |
| chr4 | 6012534 | nonsynonymous SNV | exonic |  | G | A | snp |  |
| chr4 | 6012543 | synonymous SNV | exonic |  | T | C | snp |  |
| chr4 | 6012581 | synonymous SNV | exonic |  | A | G | snp |  |
| chr4 | 6012626 | synonymous SNV | exonic |  | C | T | snp |  |
| chr4 | 6012666 | nonsynonymous SNV | exonic |  | C | T | snp |  |
| chr4 | 6012668 | synonymous SNV | exonic |  | C | G | snp |  |
| chr4 | 6013159 | nonsynonymous SNV | exonic |  | G | T | snp |  |
| chr4 | 6013168 | nonsynonymous SNV | exonic |  | G | C | snp |  |
| chr4 | 6013180 | synonymous SNV | exonic |  | C | G | snp |  |
| chr4 | 6013199 | nonsynonymous SNV | exonic |  | T | A | snp |  |
| chr4 | 6013261 | synonymous SNV | exonic |  | G | A | snp |  |
| chr4 | 6013303 | synonymous SNV | exonic |  | G | A | snp |  |
| chr4 | 6013306 | synonymous SNV | exonic |  | T | C | snp |  |
| chr4 | 6013507 | synonymous SNV | exonic |  | T | C | snp |  |
| chr4 | 6013517 | synonymous SNV | exonic |  | T | C | snp |  |
| chr4 | 6013609 | frameshift deletion | exonic |  | GGT | G | InDel |  |
| chr4 | 6013609 | frameshift deletion | exonic |  | GGT | G | InDel |  |
| chr4 | 6013612 | frameshift deletion | exonic |  | CATCGAAGGCTCCAAGGTTAACAACGTTAACGCCATGTCCGGCGGCTGTGGCTCTGGTTGTGGTGGTGGTTGCGGCAGTGGCATGG | C | InDel |  |
| chr4 | 6013612 | frameshift deletion | exonic |  | CATCGAAGGCTCCAAGGTTAACAACGTTAACGCCATGTCCGGCGGCTGTGGCTCTGGTTGTGGTGGTGGTTGCGGCAGTGGCATGG | C | InDel |  |
| chr4 | 6013707 | nonsynonymous SNV | exonic |  | G | T | snp |  |
| chr4 | 6013707 | nonsynonymous SNV | exonic |  | G | T | snp |  |
| chr4 | 6013714 | synonymous SNV | exonic;splicing |  | G | A | snp |  |
| chr4 | 6013714 | synonymous SNV | exonic;splicing |  | G | A | snp |  |
| chr4 | 6013714 | synonymous SNV | exonic;splicing |  | G | A | snp |  |
| chr4 | 6014092 | synonymous SNV | exonic |  | T | C | snp |  |
| chr4 | 6014092 | synonymous SNV | exonic |  | T | C | snp |  |
| chr4 | 6014096 | nonsynonymous SNV | exonic |  | C | T | snp |  |
| chr4 | 6014096 | nonsynonymous SNV | exonic |  | C | T | snp |  |
| chr4 | 6014128 | synonymous SNV | exonic |  | C | T | snp |  |
| chr4 | 6014128 | synonymous SNV | exonic |  | C | T | snp |  |
| chr4 | 6014149 | nonsynonymous SNV | exonic |  | G | C | snp |  |
| chr4 | 6014149 | nonsynonymous SNV | exonic |  | G | C | snp |  |
| chr4 | 6014185 | synonymous SNV | exonic |  | T | C | snp |  |
| chr4 | 6014185 | synonymous SNV | exonic |  | T | C | snp |  |
| chr4 | 6014201 | nonsynonymous SNV | exonic |  | A | G | snp |  |
| chr4 | 6014201 | nonsynonymous SNV | exonic |  | A | G | snp |  |
| chr4 | 6014206 | nonsynonymous SNV | exonic |  | C | T | snp |  |
| chr4 | 6014206 | nonsynonymous SNV | exonic |  | C | T | snp |  |
| chr4 | 6014223 | nonsynonymous SNV | exonic |  | T | C | snp |  |
| chr4 | 6014223 | nonsynonymous SNV | exonic |  | T | C | snp |  |
| chr4 | 6014266 | nonframeshift deletion | exonic |  | CGGT | C | InDel |  |
| chr4 | 6014266 | nonframeshift deletion | exonic |  | CGGT | C | InDel |  |
| chr4 | 6014312 | nonsynonymous SNV | exonic |  | A | G | snp |  |
| chr4 | 6014312 | nonsynonymous SNV | exonic |  | A | G | snp |  |
| chr4 | 6014353 | nonframeshift deletion | exonic |  | CGGT | C | InDel |  |
| chr4 | 6014353 | nonframeshift deletion | exonic |  | CGGT | C | InDel |  |
| chr4 | 6014408 | nonsynonymous SNV | exonic |  | A | C | snp |  |
| chr4 | 6014408 | nonsynonymous SNV | exonic |  | A | C | snp |  |
| chr4 | 6014437 | synonymous SNV | exonic |  | T | C | snp |  |
| chr4 | 6014437 | synonymous SNV | exonic |  | T | C | snp |  |
| chr4 | 6014455 | nonsynonymous SNV | exonic |  | C | A | snp |  |
| chr4 | 6014455 | nonsynonymous SNV | exonic |  | C | A | snp |  |
| chr4 | 6014489 | nonsynonymous SNV | exonic |  | G | A | snp |  |
| chr4 | 6014489 | nonsynonymous SNV | exonic |  | G | A | snp |  |
| chr5 | 173106393 | synonymous SNV | exonic | *Zm00001eb242900* | G | A | snp | NA |
| chr5 | 173208516 | nonsynonymous SNV | exonic | *Zm00001eb243020* | A | G | snp | NA |
| chr5 | 173208599 | nonframeshift insertion | exonic |  | A | AAAC | InDel |  |
| chr5 | 173208661 | nonframeshift insertion | exonic |  | A | AGAC | InDel |  |
| chr5 | 173208671 | nonframeshift deletion | exonic |  | TGCA | T | InDel |  |
| chr5 | 173208684 | nonframeshift insertion | exonic |  | C | CTGG | InDel |  |
| chr5 | 173208808 | nonsynonymous SNV | exonic |  | T | G | snp |  |
| chr5 | 173209146 | nonsynonymous SNV | exonic |  | T | A | snp |  |
| chr5 | 173209631 | nonframeshift insertion | exonic |  | G | GGGC | InDel |  |
| chr5 | 173209881 | synonymous SNV | exonic |  | C | T | snp |  |
| chr5 | 173209945 | nonframeshift insertion | exonic |  | T | TCCA | InDel |  |
| chr5 | 173210211 | synonymous SNV | exonic |  | C | G | snp |  |
| chr5 | 173210278 | synonymous SNV | exonic |  | C | T | snp |  |
| chr5 | 173210292 | synonymous SNV | exonic |  | C | T | snp |  |
| chr5 | 173210407 | nonsynonymous SNV | exonic |  | G | T | snp |  |
| chr5 | 173210469 | synonymous SNV | exonic |  | C | T | snp |  |
| chr5 | 173210519 | nonsynonymous SNV | exonic |  | C | G | snp |  |
| chr5 | 173210739 | synonymous SNV | exonic |  | C | T | snp |  |
| chr5 | 173210791 | nonsynonymous SNV | exonic |  | A | T | snp |  |
| chr5 | 173210894 | nonsynonymous SNV | exonic |  | C | T | snp |  |
| chr6 | 135797550 | nonsynonymous SNV | exonic | *Zm00001eb282410* | C | G | snp | BP; MF |
| chr6 | 135947299 | nonsynonymous SNV | exonic | *Zm00001eb282430* | G | C | snp | BP; MF |
| chr6 | 135947319 | nonsynonymous SNV | exonic |  | A | G | snp |  |
| chr7 | 138417957 | synonymous SNV | exonic | *Zm00001eb316700* | G | A | snp | NA |
| chr7 | 138419096 | nonsynonymous SNV | exonic |  | G | A | snp |  |
| chr7 | 138420698 | synonymous SNV | exonic |  | T | C | snp |  |
| chr7 | 138420839 | synonymous SNV | exonic |  | T | C | snp |  |
| chr7 | 138421060 | synonymous SNV | exonic |  | G | A | snp |  |
| chr7 | 138421417 | synonymous SNV | exonic |  | G | A | snp |  |
| chr7 | 138937194 | nonframeshift deletion | exonic | *Zm00001eb316880* | CGCGGCGGCGGCG | C | InDel | MF |
| chr7 | 138937194 | nonframeshift deletion | exonic |  | CGCGGCGGCGGCG | C | InDel |  |
| chr7 | 138937302 | nonsynonymous SNV | exonic |  | G | C | snp |  |
| chr7 | 138937302 | nonsynonymous SNV | exonic |  | G | C | snp |  |
| chr7 | 138937706 | nonsynonymous SNV | exonic |  | G | A | snp |  |
| chr7 | 138937706 | nonsynonymous SNV | exonic |  | G | A | snp |  |
| chr7 | 138937863 | synonymous SNV | exonic |  | C | T | snp |  |
| chr7 | 138937895 | nonsynonymous SNV | exonic |  | C | A | snp |  |
| chr7 | 138937967 | nonsynonymous SNV | exonic |  | A | C | snp |  |
| chr7 | 138938014 | nonsynonymous SNV | exonic |  | G | A | snp |  |
| chr7 | 138938274 | synonymous SNV | exonic |  | C | G | snp |  |
| chr7 | 138938339 | nonsynonymous SNV | exonic |  | C | T | snp |  |
| chr7 | 138938692 | synonymous SNV | exonic |  | A | G | snp |  |
| chr7 | 138938983 | synonymous SNV | exonic |  | C | T | snp |  |
| chr7 | 138939073 | synonymous SNV | exonic |  | C | T | snp |  |
| chr7 | 138939148 | synonymous SNV | exonic |  | G | C | snp |  |
| chr7 | 138939187 | synonymous SNV | exonic |  | C | G | snp |  |
| chr7 | 145426399 | synonymous SNV | exonic | *Zm00001eb318450* | C | A | snp | NA |
| chr7 | 145426901 | nonsynonymous SNV | exonic |  | T | C | snp |  |
| chr7 | 145428679 | nonsynonymous SNV | exonic |  | A | G | snp |  |
| chr7 | 145428726 | synonymous SNV | exonic |  | C | T | snp |  |
| chr7 | 145428736 | nonsynonymous SNV | exonic |  | G | A | snp |  |
| chr8 | 126300229 | synonymous SNV | exonic | *Zm00001eb353170* | T | C | snp | BP |
| chr8 | 126300244 | synonymous SNV | exonic |  | C | T | snp |  |
| chr8 | 126300297 | nonsynonymous SNV | exonic |  | C | T | snp |  |
| chr8 | 126300328 | synonymous SNV | exonic |  | A | C | snp |  |
| chr8 | 126300399 | synonymous SNV | exonic |  | G | A | snp |  |
| chr8 | 126300445 | synonymous SNV | exonic |  | A | G | snp |  |
| chr8 | 126300601 | synonymous SNV | exonic |  | G | A | snp |  |
| chr8 | 126300652 | synonymous SNV | exonic |  | C | T | snp |  |
| chr8 | 126300731 | nonsynonymous SNV | exonic |  | T | C | snp |  |
| chr8 | 126300734 | nonframeshift insertion | exonic |  | T | TGTG | InDel |  |
| chr8 | 126300761 | nonsynonymous SNV | exonic |  | G | A | snp |  |
| chr8 | 126301156 | synonymous SNV | exonic |  | T | G | snp |  |
| chr8 | 126301162 | synonymous SNV | exonic |  | C | T | snp |  |
| chr8 | 126301209 | nonsynonymous SNV | exonic |  | C | T | snp |  |
| chr8 | 126301246 | synonymous SNV | exonic |  | G | A | snp |  |
| chr8 | 126301251 | nonsynonymous SNV | exonic |  | G | A | snp |  |
| chr8 | 126301282 | nonsynonymous SNV | exonic |  | G | T | snp |  |
| chr8 | 126301301 | nonsynonymous SNV | exonic |  | C | T | snp |  |
| chr8 | 126301303 | synonymous SNV | exonic |  | G | A | snp |  |
| chr8 | 126301305 | nonsynonymous SNV | exonic |  | C | T | snp |  |
| chr8 | 126301324 | synonymous SNV | exonic |  | G | A | snp |  |
| chr8 | 126301404 | nonsynonymous SNV | exonic |  | C | T | snp |  |
| chr8 | 126301405 | synonymous SNV | exonic |  | G | A | snp |  |
| chr8 | 126301406 | nonsynonymous SNV | exonic |  | C | T | snp |  |
| chr8 | 126301410 | nonsynonymous SNV | exonic |  | C | T | snp |  |
| chr8 | 126301421 | nonsynonymous SNV | exonic |  | A | G | snp |  |
| chr8 | 126301423 | synonymous SNV | exonic |  | G | A | snp |  |
| chr8 | 126301425 | nonsynonymous SNV | exonic |  | A | G | snp |  |
| chr8 | 126301438 | synonymous SNV | exonic |  | C | T | snp |  |
| chr8 | 126301478 | nonsynonymous SNV | exonic |  | T | C | snp |  |
| chr8 | 126301486 | synonymous SNV | exonic |  | G | A | snp |  |
| chr8 | 126301491 | nonsynonymous SNV | exonic |  | G | A | snp |  |
| chr8 | 126301504 | nonsynonymous SNV | exonic |  | C | A | snp |  |
| chr8 | 126301520 | nonsynonymous SNV | exonic |  | G | A | snp |  |
| chr8 | 126301524 | nonsynonymous SNV | exonic |  | C | T | snp |  |
| chr8 | 126301536 | nonsynonymous SNV | exonic |  | G | T | snp |  |
| chr8 | 126301572 | nonsynonymous SNV | exonic |  | C | T | snp |  |
| chr8 | 126301581 | nonsynonymous SNV | exonic |  | C | T | snp |  |
| chr8 | 126301598 | nonsynonymous SNV | exonic |  | C | T | snp |  |
| chr8 | 126301609 | synonymous SNV | exonic |  | C | T | snp |  |
| chr8 | 126301631 | nonsynonymous SNV | exonic |  | C | G | snp |  |
| chr8 | 126301632 | nonsynonymous SNV | exonic |  | C | T | snp |  |
| chr8 | 126301645 | synonymous SNV | exonic |  | C | T | snp |  |
| chr8 | 126301663 | synonymous SNV | exonic |  | C | T | snp |  |
| chr8 | 126301671 | nonsynonymous SNV | exonic |  | C | A | snp |  |
| chr8 | 126301672 | synonymous SNV | exonic |  | C | T | snp |  |
| chr8 | 126301675 | synonymous SNV | exonic |  | G | A | snp |  |
| chr8 | 126301683 | nonsynonymous SNV | exonic |  | C | T | snp |  |
| chr8 | 126301689 | nonsynonymous SNV | exonic |  | C | T | snp |  |
| chr8 | 126301712 | nonsynonymous SNV | exonic |  | G | A | snp |  |
| chr8 | 126301720 | synonymous SNV | exonic |  | T | C | snp |  |
| chr8 | 126301740 | nonsynonymous SNV | exonic |  | G | T | snp |  |
| chr8 | 126301741 | synonymous SNV | exonic |  | C | T | snp |  |
| chr8 | 127773469 | synonymous SNV | exonic | *Zm00001eb353540* | G | A | snp | BP; MF |
| chr8 | 127773477 | nonframeshift insertion | exonic |  | G | GGCACAC | InDel |  |
| chr8 | 127773520 | synonymous SNV | exonic |  | G | C | snp |  |
| chr8 | 127773537 | nonsynonymous SNV | exonic |  | T | C | snp |  |
| chr8 | 127773564 | nonsynonymous SNV | exonic |  | A | G | snp |  |
| chr8 | 127773775 | synonymous SNV | exonic |  | C | G | snp |  |
| chr8 | 127773801 | frameshift deletion | exonic |  | GTGCAT | G | InDel |  |
| chr8 | 127773863 | stopgain SNV | exonic |  | T | TGCTA | InDel |  |
| chr8 | 127774039 | nonsynonymous SNV | exonic |  | C | G | snp |  |
| chr9 | 51588414 | nonsynonymous SNV | exonic | *Zm00001eb382210* | G | A | snp | NA |
| chr9 | 51588436 | synonymous SNV | exonic |  | C | A | snp |  |
| chr9 | 51588440 | nonsynonymous SNV | exonic |  | C | T | snp |  |
| chr9 | 51588441 | nonsynonymous SNV | exonic |  | G | A | snp |  |
| chr9 | 51588453 | nonsynonymous SNV | exonic |  | C | T | snp |  |
| chr9 | 51588458 | nonsynonymous SNV | exonic |  | T | C | snp |  |
| chr9 | 51588470 | nonsynonymous SNV | exonic |  | A | C | snp |  |
| chr9 | 51588474 | nonsynonymous SNV | exonic |  | C | T | snp |  |
| chr9 | 51588495 | nonsynonymous SNV | exonic |  | A | G | snp |  |
| chr9 | 51588498 | nonsynonymous SNV | exonic |  | C | T | snp |  |
| chr9 | 51588499 | synonymous SNV | exonic |  | C | T | snp |  |
| chr9 | 51588503 | nonsynonymous SNV | exonic |  | G | A | snp |  |
| chr9 | 51588509 | nonsynonymous SNV | exonic |  | C | T | snp |  |
| chr9 | 51588516 | nonsynonymous SNV | exonic |  | A | C | snp |  |
| chr9 | 51588518 | nonsynonymous SNV | exonic |  | G | A | snp |  |
| chr9 | 51588541 | synonymous SNV | exonic |  | A | G | snp |  |
| chr9 | 51588582 | nonsynonymous SNV | exonic |  | C | T | snp |  |
| chr9 | 51591133 | nonsynonymous SNV | exonic |  | G | A | snp |  |
| chr9 | 51591148 | nonsynonymous SNV | exonic |  | A | T | snp |  |
| chr9 | 51591864 | synonymous SNV | exonic |  | G | A | snp |  |
| chr9 | 51591874 | nonsynonymous SNV | exonic |  | T | A | snp |  |
| chr9 | 51591885 | nonsynonymous SNV | exonic |  | C | G | snp |  |
| chr9 | 51591890 | nonsynonymous SNV | exonic |  | G | T | snp |  |
| chr9 | 51592012 | nonsynonymous SNV | exonic |  | C | G | snp |  |
| chr9 | 51592064 | stopgain SNV | exonic |  | G | A | snp |  |
| chr9 | 51592071 | synonymous SNV | exonic |  | A | T | snp |  |
| chr9 | 51592116 | synonymous SNV | exonic |  | T | G | snp |  |
| chr9 | 51592118 | nonsynonymous SNV | exonic |  | A | G | snp |  |
| chr9 | 51592251 | nonsynonymous SNV | exonic |  | C | T | snp |  |
| chr9 | 51592525 | nonsynonymous SNV | exonic |  | A | C | snp |  |
| chr9 | 51593012 | nonsynonymous SNV | exonic |  | G | C | snp |  |
| chr9 | 51593059 | nonsynonymous SNV | exonic |  | C | G | snp |  |
| chr9 | 51593072 | synonymous SNV | exonic |  | C | A | snp |  |
| chr9 | 51593090 | synonymous SNV | exonic |  | C | T | snp |  |
| chr9 | 51593117 | synonymous SNV | exonic |  | T | C | snp |  |
